# Mechanism of DAPK1 for Regulating Cancer Stem Cells in Thyroid Cancer

**DOI:** 10.3390/cimb46070422

**Published:** 2024-07-05

**Authors:** Mi-Hyeon You

**Affiliations:** Department of Anatomy, Konkuk University College of Medicine, 50-1, 268 Chungwon-daero, Cungju-si 27478, Republic of Korea; shineth@kku.ac.kr; Tel.: +82-43-840-3722

**Keywords:** death-associated protein kinase 1 (DAPK1), cancer stem cell (CSC), epithelial to mesenchymal transition (EMT), papillary thyroid carcinoma (PTC)

## Abstract

Death-associated protein kinase 1 (DAPK1) is a calcium/calmodulin (Ca^2+^/CaM)-dependent serine/threonine (Ser/Thr) protein kinase and is characteristically downregulated in metastatic cancer. Several studies showed that DAPK1 is involved in both the early and late stages of cancer. DAPK1 downregulation is elaborately controlled by epigenetic, transcriptional, posttranscriptional, and posttranslational processes. DAPK1 is known to regulate not only cancer cells but also stromal cells. Recent studies showed that DAPK1 was involved not only in tumor suppression but also in epithelial-mesenchymal transition (EMT) and cancer stem cell (CSC) formation in colon and thyroid cancers. CSCs are major factors in determining cancer aggressiveness in cancer metastasis and treatment prognosis by influencing EMT. However, the molecular mechanism involved in the regulation of cancer cells by DAPK1 remains unclear. In particular, little is known about the existence of CSCs and how they are regulated in papillary thyroid carcinoma (PTC) among thyroid cancers. In this review, we describe the molecular mechanism of CSC regulation by DAPK1 in PTC progression.

## 1. Introduction

Metastasis is achieved through invasion, intravasation, circulation, extravasation, and the colonization of primary cancer. Although less than 0.1% of tumor cells metastasize in melanoma, it represents the main cause of cancer treatment failure [1,2,3]. Epithelial-mesenchymal transition (EMT) and cancer stem cells (CSC) play important roles when invasion and metastatic activation occur [4]. Fibroblasts and hypoxia in metastasized areas were recently reported to promote signaling to facilitate metastasis [2,5,6,7].

DAPK1 is a serine/threonine (Ser/Thr) kinase with a death domain that is regulated by calcium/calmodulin (Ca^2+^/CaM) [8,9,10]. DAPK1 known to regulate several stress-induced cell death pathways. Endoplasmic reticulum (ER) stress signaling induces cell death in two directions: caspase-dependent apoptosis (type I cell death) and autophagy (type II autophagy) [11,12]. DAPK1 acts as a tumor suppressor and controls tumor growth in the early stages. However, in later stages, such as in colon and thyroid cancer, it is involved in the expression of tumor EMT and stem cells. One study focusing on breast cancer showed that DAPK1 inhibited the function of Pin1 isomerase by phosphorylating Ser71 in the catalytic activity site [13]. In that study, DAPK1 was shown to inhibit the centrosome amplification and cell transformation of cancer cells, and a negative correlation was demonstrated with Pin1, which is an oncogene in breast cancer [13].

DAPK1 is abundant in developing brain tissue [14] and gradually decreases after birth. However, it is also highly expressed in the cerebral cortex and hippocampus in adult rats and mice [14]. Several studies have shown that both DAPK1 knockout and the inhibition of DAPK1 function protected the brain and, conversely, that the overexpression of DAPK1 had various adverse effects on the brain [15,16,17]. Many studies have indicated that DAPK1 might play an important role in various acute and chronic neurological diseases, such as Alzheimer’s disease (AD), Parkinson’s disease, Huntington’s disease, traumatic brain injury, and stroke [12,15,16,17,18,19,20,21,22,23,24,25,26].

The current review focuses on the role of DAPK1 in cancer cells, and particularly the role that DAPK1 plays in papillary thyroid carcinoma (PTC) cells during metastasis [27].

## 2. DAPK1 Properties and Roles in Cancer

### 2.1. DAPK1 Structure

**Figure 1:**
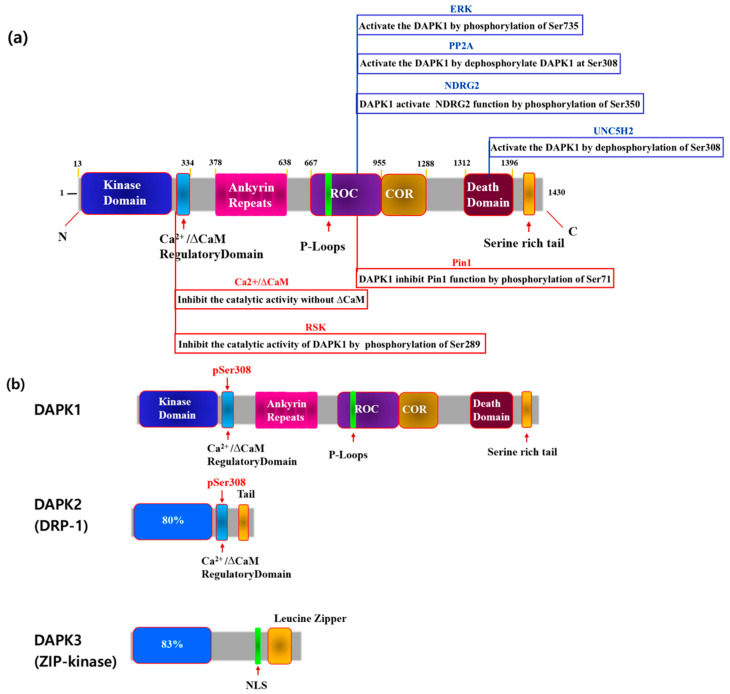
Schematic depiction of the multi-domain organization of the (DAPK1) protein. (**a**) The size of the amino acid regions of DAPK1 and the interacting proteins in each region are shown in the diagram. The blue protein is an interactor that activates the function of DAPK1, and the red protein is an interactor that inhibits it. The blue protein is indicated above the DAPK1 amino acid sequence, and the red protein is indicated below. (**b**) Schematic diagram of the DAPK family.

DAPK1 was first reported as a tumor suppressor in 1995. Since then, three homologous human DAPK families with catalytic domains different from DAPK1 have been reported [8,28]. Among them, DAPK1 consists of 1431 amino acids with a Ras of the complex (ROC) domain, a death domain, and a Ser-rich C-terminal tail [9,28]. The activity of DAPK1 has been shown to be negatively regulated by Ser308 autophosphorylation at the calmodulin (ΔCaM) site (Figure 1) [9,28].

DAPK1 is a 1431-amino acid kinase consisting of a Ca^2+^/CaM-binding autoregulatory domain, eight ankyrin repeats, putative P-loops, a ROC domain, a C-terminal of the ROC (COR) domain, a death domain, and a serine-rich C-terminal tail [28,29,30], (Figure 1). Each of these domains plays a variety of roles, such as catalysis, degradation, and localization, through binding processes with other substrates.

Among them, the Ca^2+^/CaM autoregulation domain regulates the activity of DAPK1 through CaM binding and is regulated by Ca^2+^ binding (Figure 1). In the absence of CaM, the autoregulatory domain binds to the catalytic domain and inhibits DAPK1 function by blocking other substrates from binding to DAPK1 (Figure 1). Therefore, mutant DAPK1 with a deletion of this part is always active [31]. The Ca^2+^/CaM autoregulatory domain is also known to regulate DAPK1 activity by allowing its phosphorylation (Figure 1). DAPK1 inhibits catalytic activity by autophosphorylating Ser308 in this region. The phosphor-Ser residue of DAPK1 forms a negative charge, thus allowing for additional contact between the CaM autoregulatory domain and the catalytic cleft (Figure 1). Phosphorylated DAPK1 also has a low affinity for CaM. PP2A is known to be a phosphatase that activates DAPK by dephosphorylating Ser308 (Figure 1) [32].

DAPK1 has 10 ankyrin repeats, and the degradation of DAPK1 can be observed in this region through the ubiquitin–proteasome pathway. For example, DAPK1-1-interacting protein (DIP-1) ubiquitinates DAPK1 at this site, thus promoting degradation [33]. Through this domain, DAPK1 is known to activate LAR by dephosphorylating Tyr491/492 [31]. DAPK1 has two p-loops, 639–646 and 695–702 (Figure 1). GTP (Guanosine-5′-triphosphate) binding occurs at this point [31].

The ROC-COR domain exists between residues 667–1288 of DAPK1, where it partially overlaps some cytoskeletal locations (Figure 1). DAPK1 binds to leucine repeat-rich kinase 2 (LRRK2) at this site, which is another ROCO family protein [31]. Pin1 is also phosphorylated at Ser71 by DAPK1 at this site, thus limiting its role [13,31]. The subsequent amino acid residues 667–1228 are part of the cytoskeletal domain. DAPK1 binds to the N-myc downstream-regulated gene 2 (NDRG2) at this site and phosphorylates Ser350 to induce neuronal cell death [12,31] (Figure 1).

The death domain of DAPK1 is located between residues 1312 and 1396 and regulates protein–protein interactions, kinase activity, and apoptosis. This site reacts with extracellular signal-regulated kinase (ERK), which phosphorylates Ser735 at this site in DAPK1 to increase catalytic activity [34]. DAPK1 is also known to regulate tau phosphorylation by interactions between microtubule affinity-regulating kinases (MARK1/2) and its death domain [33]. Thus, DAPK1 is believed to regulate microtubule (MT) destabilization and tau toxicity through this region [33]. DAPK1 also binds to Pin1 isomerase (peptidyl-prolyl cis-trans isomerase) at this site and phosphorylates Pin1 at Ser71, inhibiting its function [13]. Pin1 is a phosphorylation-specific prolyl isomerase that regulates numerous signaling molecules that promote tumor growth in cancer. DAPK1 inhibits the ability of Pin1 to induce phagosome amplification and cell transformation in breast cancer. Both in vitro and in vivo studies have also proven that the function of Pin1, which acts in a neuroprotective manner by suppressing tau phosphorylation, is also regulated through this region [35]. Pin1 is expressed to varying degrees in cancer, and the binding of the death domain of DAPK1 to Pin1 inhibits the function of Pin1.

DAPK1 increases the activity of pyruvate kinase M2 (PKM2, a key glycolytic enzyme) through binding to death domain thus increasing both the glycolysis rate and lactate production [35]. Notably, MARK 1/2, Pin1, and PKM2 all regulate the activating effect of DAPK1 through binding to the death domain. TSC2 forms a TSC1/TSC2 dimer and activates the mechanistic target of rapamycin (mTOR) [36]. DAPK1 interferes with the formation of this dimer and ultimately inhibits mTOR1 signaling, thus causing autophagy and apoptosis.

### 2.2. Death-Associated Protein Kinase Family

DAPK1 was first identified to cause interferon (IFN)-mediated cell death through screening an anti-sense cDNA library in HeLa cells [10]. Since then, four additional kinases with some degree of homology to the catalytic domain of DAPK1 have been identified. Among them, DAPK2 (DRP-1) is the domain most similar to DAPK1. DAPK1, DAPK2, and DAPK3 (ZIP-Kinase) can all be classified as common kinases because their catalytic domains are located at the N-terminus (Figure 1b). DAPK1 is a 160 kDa protein kinase that is regulated by Ca^2+^/CaM. In comparison, DRAK1 is 55 kDa, while DAPK2 is relatively small at 42 kDa and does not have a Ca^2+^/CaM domain. However, studies have shown that it has a leucine zipper structure along with a nuclear localization signal at the C-terminus [26,32]. DAPK3 also has a different structure (Figure 1). Both DAPK and DRP-1 possess a Ca^2+^/CaM autoregulatory domain C-terminus that is highly homologous to the catalytic domain (residues 278–320 of DAPK). DAPK and DRP-1 both autophosphorylate Ser308 within the Ca^2+^/CaM autoregulatory domain as a mechanism to inhibit catalytic activity. In cerebral neurons, in particular, calcium-activated calcineurin has been identified as a phosphatase that dephosphorylates Ser308 in response to ischemic injury [26]. In summary, two events are required to activate DAPK and DRP-1. One, which is common to all CaM-dependent kinases, is the binding of Ca^2+^-activated CaM to an autoregulatory CaM-binding moiety. The other, which is unique to DAPK and DRP-1, is the dephosphorylation of Ser308, which increases affinity for CaM.

### 2.3. Phosphorylation of DAPK1

DAPK1 is phosphorylated by several different substrates, and its function is regulated (Figure 1). Figure 1 shows which DAPK1 structure of each substrate it attaches to and acts on and those that enhance the function of DAPK1, including NDRG2, protein phosphatase 2A (PP2A), p90 ribosomal S6 kinases 1/2 (RSK 1/2), UNC5H2, and ERK. Pin1 is another substance that inhibits DAPK1 function (Figure 1).

Several dephosphorylated phosphatases that regulate DAPK1 have been reported, among which PP2A is a well-known example [29]. PP2A dephosphorylates DAPK1 at Ser308, activating DAPK1 and thereby inducing autophagy, cell proliferation, and cell apoptosis in vitro [11]. A previous study found that UNC5H2 binds to the death domain of DAPK1 and activates it by dephosphorylating Ser308 in DAPK1. In this way, it activates the apoptotic function of DAPK1 [29]. The phosphorylation of Ser735 in DAPK1 by ERK can result in cell apoptosis [30], and the phosphorylation of Ser289 in DAPK1 by RSK can reduce cell apoptosis [31]. DAPK1 also regulates brain aging by phosphorylating Tau and APP, as well as the isomerase Pin1. Phospho-peptide library screening showed that DAPK1 phosphorylates N-myc downstream regulatory gene 2 (NDRG2) [12,18,19]. DAPK1 can directly phosphorylate Ser350 in NDRG2, and the NDRG2 domain can specifically bind to DAPK1 through the ROC-COR domain [12]. NDRG2 has been shown to promote the death of neurons in a mitochondria-dependent manner in both in vitro and in vivo human samples [12]. NDRG2 is overexpressed in AD. Since NDRG2 was originally known as a tumor suppressor, additional studies are needed to determine the role that Ser350 in NDRG2 plays in cancer metastasis.

### 2.4. DAPK1 Is a Tumor Suppressor

DAPK1 induces apoptosis. DAPK1 is associated with the cytoskeleton. In 1997, DAPK1 was cloned, and its tumor-suppressing ability was tested in two murine lung tumors through in vivo experiments that featured low expression levels of DAPK1 but the ability to metastasize [10,31]. DAPK1 was restored to some extent in Lewis carcinoma with high metastatic potential. The authors of that study injected DAPK1-transfected cells into the tail vein [10]. Local tumor growth was delayed at the site of secondary metastasis. Terminal deoxynucleotidyl transferase dUTP nick end labeling (TUNEL) staining of metastasized tumor sections revealed that DAPK expression enhanced apoptosis in vivo. These results suggest that DAPK1 is involved in the multi-step metastasis process, specifically by making tumor cells sensitive to apoptosis during cancer metastasis. DAPK1 has also been shown to play a role in a metastasized microenvironment [37,38,39].

DAPK1′s regulation of metastasis has been studied in several cancers, including colon, lung, and thyroid cancers [27,40,41]. In colon cancer, hypoxic conditions increased the expression of miR 103/107 while decreasing the expressions of both DAPK1 and KLF4, thereby promoting cancer metastasis [40]. The results of a previous study from our laboratory showed that the downregulation of DAPK1 could promote the metastatic stage of thyroid cancer [27].

The downregulation or inactivation of DAPK1 in pituitary tumors and liver cancer was shown to promote cancer progression or make it more aggressive [42,43]. Therefore, DAPK1 can not only act as a tumor suppressor but also promote metastasis.

### 2.5. Role of DAPK1 in Cancer Metastasis

Although metastasis is an important feature of malignant tumors and a major cause of cancer treatment failure, the process by which it occurs is yet to be elucidated [44,45,46].

A recent study found that the level of the tumor suppressor DAPK1 is regulated in several ways during cancer metastasis [31]. Initially, the downregulation or inactivation of DAPK1 can regulate the early stages of cancer proliferation as a tumor suppressor [9]. At this time, DAPK1 in the cytosol can regulate cytoskeletal proteins to suppress cell morphological changes or motility [44]. DAPK1 induces an imbalance between cell adhesion and cell adhesion, which regulates cell motility and the cytoskeleton by promoting cell separation from the extracellular matrix. DAPK1-mediated integrin inactivation not only disrupted cell polarity by blocking the activation of Cdc42 GTPase but also decreased cell persistence and inhibited cell migration [47]. Another pathway induces the phosphorylation of MLCII at Ser19 [31], with the intended effect of increasing actomyosin contractility. This contractility contributes to membrane bleb formation and induces cell death. MAP1B interacts through the kinase domain of DAPK1 and participates in microtubules, thus contributing to membrane bleb formation [31]. DAPK1 has also been reported to interact with the F-actin regulators LIMK and cofilin [31]. Cofilin depletion increased actomyosin assembly and myosin II activity, ultimately leading to membrane blabbing.

Significant hypermethylation has been observed near the DAPK1 promoter in 20 cancer types [48]. The 5′-UTR of the DAPK1 gene contains a CpG island whose hypermethylation causes gene silencing. A previous study on thyroid cancer reported that the hypermethylation of DAPK1 in papillary carcinoma significantly increased the aggressiveness (metastasis and lymph node metastasis) of thyroid carcinoma [49]. In addition to hypermethylation, the downregulation of DAPK1 is known to be dependent on microRNA [50]. The significant downregulation of DAPK1 in colon cancer is associated with miR103 and miR107, which are closely related to patient prognosis [50,51]. In metastatic tumors, DAPK1 expression is also regulated at the transcriptional level. The transcription factor C/EBP-beta is known to regulate DAPK1 levels induced by IFN-r [52].

In cancer progression, the mechanism by which DAPK1 inhibits tumor metastasis as a tumor suppressor involves various apoptotic signals in caspase-dependent apoptosis and caspase-independent autophagy [33]. To elaborate, DAPK1 controls tumor cell motility and invasion through autophagy, metastatic potential for anoikis, and escape from immune surveillance. One of the important targets of DAPK1 is the tumor suppressor p53. DAPK1 induces p53 through two main mechanisms. First, DAPK1 stabilizes and activates p53 by inactivating Mouse double minute 2 homolog (MDM2, E3 ubiquitin–protein ligase) which is achieved by inducing p19ARK [51]. Another mechanism is the inactivation of integrin. This not only induces the inactivation of FAK and the upregulation of p53 but also inhibits cell–matrix adhesion, which ultimately induces cell apoptosis [51]. Other targets of DAPK1 are ERK1 and ERK2, which are both known to bind to DAPK1, as well as promote its phosphorylation and, thus, its catalytic activity [16]. ERK1 and 2 promote DAPK1-mediated apoptosis. DAPK1 also blocks the nuclear translocation of ERK [16], which blocks cell proliferation signaling by ERK. This interaction mediates cell apoptosis rather than survival.

During metastasis, cancer cells have to survive in new environments, such as those with hypoxia, new microenvironments, and nutrient deprivation [4]. The tumor microenvironment is composed of various cell types, such as cancer-associated fibroblasts, endothelial cells, immune cells, and stromal cells. These cells provide a microenvironment environment that allows tumors to grow [4]. Cancer cells have to adapt to new environments through EMT, increased CSC expression, increased expression of the heme oxygenase inducible factor 1 (HIF1)-α pathway during hypoxia, and interactions with fibroblasts [4,28,45,53]. A recent study showed that DAPK1 regulates cancer stem cells in prostate cancer by interacting with the zinc finger E-box-binding homeobox-1 (ZEB1) to inhibit the Hippo/YAP signaling pathway in PCa-CD133(+) cells [54]. That study found that DAPK1 could inhibit ZEB1 expression in PCa mouse tumor tissues [54]. In thyroid cancer, which is discussed in further detail later, DAPK1 is believed to have a close relationship with OCT4 (POU5F1), the master gene that controls CSC stemness. This means that DAPK1 can act as an early tumor suppressor and also closely interact with CSCs and EMT regulators, which are prognostic markers of cancer metastasis. How DAPK1 regulates CSCs and EMT in various cancer metastasis processes should be studied further. The results obtained to date indicate the potential of DAPK1 as a regulator of cancer metastasis.

DAPK1 also affects tumor-associated vasculature, which is an important factor in the microenvironment. Netrin-1 receptor, unc-5 netrin receptor B (UNC5H2/B), is a cell death-dependent receptor that is triggered by DAPK1 in endothelial cells, thus causing apoptosis. UNC5H2/B attaches to the death domain of DAPK1 and inhibits the autophosphorylation of Ser308, thereby inhibiting DAPK1 catalytic activity by interfering with CaM. Netrin-1 binds to UNC5H2/B and inhibits this apoptotic effect, increasing the survival of endothelial cells. Studies have found that Netrin-1 is upregulated in lung cancer and breast cancer that metastasize easily [32].

### 2.6. Role of DAPK1 in Thyroid Cancer

**Figure 2:**
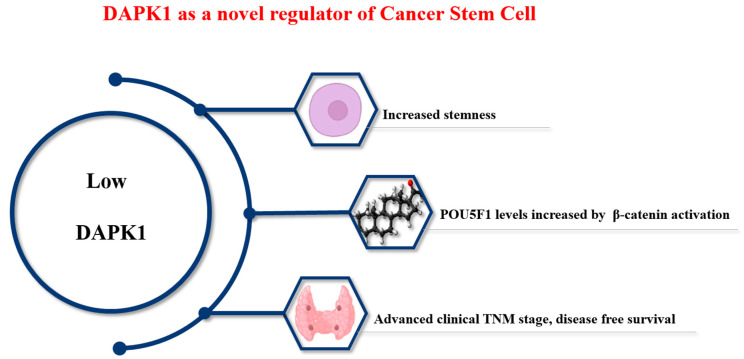
Role of DAPK1 in cancer stem cell progression.

Thyroid cancer is one of the most common cancers [55,56]. Unlike other cancers, the prognosis of patients with thyroid cancer is good. Treatment typically involves a combination of surgery, radiation therapy, and hormone therapy. However, distant metastasis occurs in 5–10% of cases [27], and prognosis is poor when distant metastasis occurs [27]. CSCs are involved in recurrent metastasis and the treatment resistance of cancer cells [57,58,59], as shown in Figure 2.

Studies on CSCs in thyroid cancer reported that CSCs could progress in several directions [60,61]. Although CSCs have been reported in many cancers, such as breast cancer, colorectal cancer, stomach cancer, and thyroid cancer, their mechanism of action is not well known [54,60,61,62]. Isolated CSCs were shown to promote tumor formation in immunodeficient mice [54]. EMT, which is a characteristic of thyroid cancer with poor prognosis, and drug-resistant genes, ATP-binding cassette super-family G member 2 (ABCG2), has also been reported to be associated with CSCs [62].

A previous thyroid cancer study indicated the mechanism by which DAPK1 regulates OCT4 (POU5F1), which is a master gene that determines CSC properties (Figure 2) [27]. That study examined the mRNA expression of DAPK1 in thyroid cancer and found that the expression of DAPK1 in tumor tissue was remarkably low. The dataset was then divided into two parts to investigate the relationship between DAPK1 expression and stemness, and a relatively high stemness index was observed in tumor tissue with low DAPK1 expression. Considering that the low DAPK1 tumor group showed high levels and aggressiveness in tumor metastasis. The findings indicated an indirect association between DAPK1 and CSCs. After the overexpression of DAPK1 in 8505c [27,62], MDA-T32, and TPC-1 cell lines, tumorsphere analysis (100 nm) and Aldefluor analysis at 10 days confirmed that DAPK1 was a negative regulator of thyroid cancer stemness [27,62].

Finally, we investigated stemness according to the protein expression of DAPK1 in 143 human PTC samples. Among the 143 PTC samples examined, 101 (71%) were found to be positive for DAPK1. Characteristically, lower DAPK1 expression was observed in both T3-4 PTC and lymph node (LN) metastasis. Significant correlations with Oct, Sox, and Nanog expression were seen in Western blots performed using the same samples. These results indicate that DAPK1 can regulate cancer progression by regulating CSCs in the progression of thyroid cancer not only in cell lines but also in human tissue [27,61,62].

DAPK1 was found to regulate the level of Oct4 through the β-catenin pathway both in vitro and in vivo using human PTC samples. The downregulation of DAPK1 significantly increased β-catenin in the nucleus, which is consistent with the findings of previous studies [58,59]. Our results suggest that DAPK1 can not only act as a tumor suppressor when the prognosis of PTC cancer is poor, but it can also affect CSC formation [59].

The most well-known DAPK1-induced PTC study is a thesis reporting that the hypermethylation of DAPK1 has significant effects (metastasis, lymph node metastasis) in thyroid carcinoma [27,62]. The results of our study are consistent with these studies [27]. DAPK1 hypermethylation (inactivation) and DAPK1 downregulation during the process of metastasis are believed to be meaningful because they link CSCs with cancer aggressiveness [49]. However, the findings in this study were insufficiently verified using in vivo experiments [27]. Thus, additional research is needed.

### 2.7. Evaluating DAPK as a Therapeutic Target

Because regulating DAPK1 expression can affect cancer progression, studies have examined the activation of DAPK1. The activation of DAPK1 is achieved by increasing DAPK1 mRNA transcription, upregulating protein expression, or activating the Ser 308 autophosphorylation of DAPK1. Currently, nine substances are known to increase the expression of DAPK1, resulting in increased mRNA.

5-Aza-20-deoxycytidine, a demethylation agent, inhibited cell growth or induced apoptosis by increasing DAPK1 mRNA levels in cancer cell lines [63]. Curcumin increased both mRNA and the protein expression of DAPK1 in the U251 cell line, causing G2/M arrest and apoptosis [64]. Sodium selenite regulated DAPK1 expression (mRNA, protein, Ser308 dephosphorylation) in the HL60 cell line and induced apoptosis through the autophagy pathway [65]. Dual panobinostat/LBH589 (histone deacetylation inhibitor) induced autophagy by dephosphorylating DAPK1 in HCT116 cells [66]. Trichostatin A (histone deacetylation) increased DAPK1 protein expression in the A549 cell line, ultimately causing cell death [67]. Although studies have investigated the mechanisms of these various reagents, no drug that specifically and directly activates DAPK1 kinase has been reported. Even if DAPK1 activity is activated, it is difficult to know whether only DAPK1 is activated and whether it proceeds through the general regulatory proteasomal degradation pathway Curcumin is a functional DAPK1 activator, and additional research should be conducted to determine whether other drugs activate only DAPK1 alone, using curcumin as a control. Moreover, since DAPK1 binds to binding partners in multiple domains and regulates cellular function, additional research that focuses on the synergistic effects of administering them together is needed (Figure 1).

## 3. Conclusions

DAPK1 was first identified as a tumor suppressor gene. Since then, several studies have examined the structure of DAPK1, and substrates that interact with DAPK1 have been discovered (Figure 1 and Figure 2). Among the various roles played by DAPK1, DAPK1 has been recently shown to be involved in the cancer metastasis process in various ways. In this review, we summarize how DAPK1 can regulate CSCs in tumor cells and influence cancer aggressiveness while focusing on thyroid cancer. We also present several studies on DAPK1 activation. These findings are expected to contribute to a better understanding of the multiple roles played by DAPK1, suggesting a new area for cancer research.

Low DAPK1 levels promote stemness in thyroid cancer. Low DAPK1 directly regulates the level of β-catenin, thereby increasing the level of OCT4 (POU5F1), the main factor that determines CSCs. This indicates a close relationship of DAPK1 with the aggressiveness of PTC cancer (tumor size, lymph node metastasis, and treatment-refractory) and suggests DAPK1 as a therapeutic target and an independent prognostic marker in thyroid cancer.

## Data Availability

The author confirms that the data supporting the findings of this study are available within the article. Additional raw data supporting the findings of this study are available from the corresponding authors (M.H.Y.) upon request.
